# Tools and best practices for data processing in allelic expression analysis

**DOI:** 10.1186/s13059-015-0762-6

**Published:** 2015-09-17

**Authors:** Stephane E. Castel, Ami Levy-Moonshine, Pejman Mohammadi, Eric Banks, Tuuli Lappalainen

**Affiliations:** New York Genome Center, New York, NY USA; Department of Systems Biology, Columbia University, New York, NY USA; Broad Institute, Cambridge, MA USA

## Abstract

Allelic expression analysis has become important for integrating genome and transcriptome data to characterize various biological phenomena such as *cis*-regulatory variation and nonsense-mediated decay. We analyze the properties of allelic expression read count data and technical sources of error, such as low-quality or double-counted RNA-seq reads, genotyping errors, allelic mapping bias, and technical covariates due to sample preparation and sequencing, and variation in total read depth. We provide guidelines for correcting such errors, show that our quality control measures improve the detection of relevant allelic expression, and introduce tools for the high-throughput production of allelic expression data from RNA-sequencing data.

**Electronic supplementary material:**

The online version of this article (doi:10.1186/s13059-015-0762-6) contains supplementary material, which is available to authorized users.

## Background

**Fig. 1:**
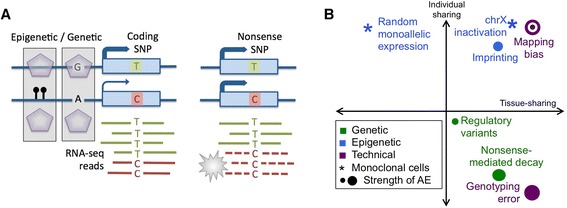
Allelic expression and its sources. **a** Schematic illustration of AE. **b** Biological sources of AE, with the x-axis denoting the approximate sharing of AE across tissues of an individual, and the y-axis having the estimated sharing of AE signal in one tissue across different individuals [5, 8, 12, 13, 15]. *SNP* single-nucleotide polymorphism

Integrating genome and transcriptome data has become a widespread approach for understanding genome function. Allelic expression (AE; also called allele-specific expression or allelic imbalance) analysis is becoming an increasingly important tool for this, as it quantifies expression variation between the two haplotypes of a diploid individual distinguished by heterozygous sites (Fig. 1a). This approach can be used to capture many biological phenomena (Fig. 1b): effects of genetic regulatory variants in *cis* [1–8], nonsense-mediated decay triggered by variants causing a premature stop codon [9–12], and imprinting [13, 14]. Standard RNA-sequencing (RNA-seq) data capture AE only when higher expression of one parental allele is shared between individual cells (Additional file 1), as opposed to random monoallelic expression of single cells that typically cancels out when a pool of polyclonal cells is analyzed [15, 16].

In this paper, we describe a new tool in the Genome Analyzer Toolkit (GATK) software package for efficient retrieval of raw allelic count data from RNA-seq data, and analyze the properties of AE data and the sources of errors and technical variation, with suggested guidelines for accounting for them. While most types of errors may be rare, they are easily enriched among sites with allelic imbalance, and can sometimes mimic the biological signal of interest, thus warranting careful analysis. Our focus is on methods for obtaining accurate data of AE rather than building a graphical user interface (GUI) pipeline [17] or downstream statistical analysis of its biological sources [9, 13, 18–20]. The example data in most of our analysis are the open-access RNA-seq data set of the lymphoblastoid cell lines (LCLs) of 1000 Genomes individuals from the Geuvadis project [5].

## Results and discussion

### Unit of AE data

**Fig. 2:**
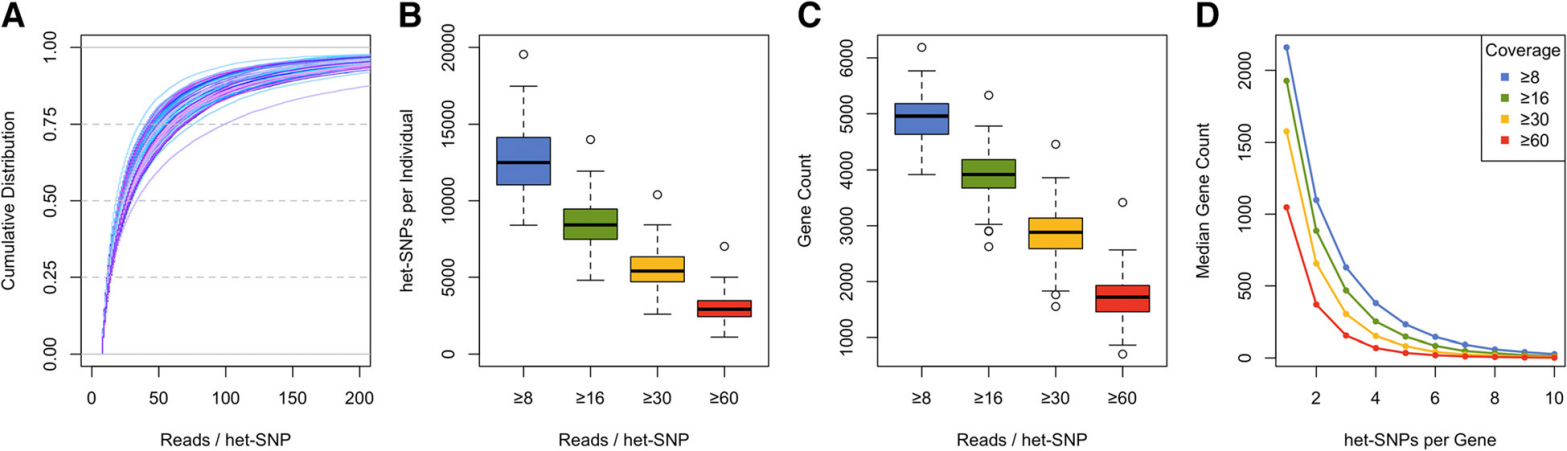
Genomic coverage of AE data in Geuvadis CEU samples. **a** Cumulative distribution of RNA-seq read coverage per het-SNP (each line represents one sample). **b**, **c** The number of het-SNPs (**b**) and protein-coding genes (**c**) per sample as a function of coverage cutoff. **d** The number of protein-coding genes with AE data versus the number of het-SNPs they contain. Each line is the median for all samples at a specific coverage level

The biological signal of interest in AE analysis is the relative expression of a given transcript from the two parental chromosomes. Typical AE data seek to capture this by counts of RNA-seq reads carrying reference and alternative alleles over heterozygous sites in an individual [heterozygous single-nucleotide polymorphisms (het-SNPs)], and this is the focus of our analysis unless mentioned otherwise. The Geuvadis samples with a median depth of 55 million mapped reads have about 5000 het-SNPs covered by ≥30 RNA-seq reads, distributed across about 3000 genes and 4000 exons (Fig. 2; Additional file 2). The exact number varies due to differences in sequencing depth, its distribution across genes, and individual DNA heterozygosity. About one half of these genes contain multiple het-SNPs per individual, which could be aggregated to better detect AE across the gene (Fig. 2d). However, alternative splicing can introduce true biological variation in AE in different exons, and incorrect phasing needs to be accounted for in downstream analysis [13]. Additionally, summing up data from multiple SNPs is not appropriate if the same RNA-seq reads overlap both sites. In the Geuvadis data, 9 % of the reads used in AE analysis in fact overlap more than one het-SNP (Figure S2d in Additional file 2), but this will become more frequent as read lengths increase [21]. In the future, better tools are needed to partition RNA-seq reads to either of the two haplotypes according to all het-SNPs that they overlap [22]. In fact, this could help to phase exonic sites separated by long introns.

AE analysis of small insertions or deletions (indels) has proven to be technically very challenging and it is rarely attempted even though frameshift indels are an important class of protein-truncating variant. Alignment errors over indel loci are pervasive due to multiple mismatches of reads carrying alternative alleles, and lower genotyping quality adds further error [12]. In Rivas et al. [12] we describe the first approach for large-scale analysis of AE over indels, but further methods development is warranted for better sensitivity and computational scalability.

In addition to classical AE analysis to detect differences in total expression level of two haplotypes, it is also possible to analyze allelic differences in transcript structure or splicing [allelic splicing (AS)] [5, 21]. These methods compare the exon distribution of reads and their mates carrying different alleles of a heterozygous site, and work increasingly well for longer total fragment lengths. In these analyses, the data structure is somewhat more complex than reference/non-reference read counts in AE, depending on the specific algorithm. While this paper focuses on classical AE analysis of SNPs, most of the quality analysis steps apply to indel AE and AS analyses as well.

### Tools to retrieve allele counts

Allele counts are the starting point for all AE analyses, and many previous tools can retrieve these counts. However, they also perform other analyses that require additional input data and increase the runtime. Here we present simple tools that can be used to retrieve only allele counts, using the minimum required inputs in standard formats. We present two solutions: 1) a highly efficient Python tool that processes results from SAMtools mpileup, the framework used by the majority of existing AE analysis pipelines; and 2) an easy to use tool in the widely used GATK v.3.4 [23, 24] called ASEReadCounter, which does not require any additional setup, and includes a variety of easily customizable read processing options as well as professional maintenance and documentation, similar to other GATK tools. Both operate on aligned RNA-seq reads and count the reference and alternative allele reads that passed filters for mapping and base quality at each bi-allelic heterozygous variant. The GATK tool offers several additional options for processing RNA-seq reads: by default each read fragment is counted only once if the base calls are consistent at the site of interest, and duplicate reads are filtered (see below). Other options allow filtering for coverage and for sites or reads with deletions. The output of both is one file per RNA-seq input file, with one line per site displaying the counts for each allele as well as counts of filtered reads, and can be used for downstream analyses. The tools yield consistent results, with runtimes comparable to a previously published tool [25] (Additional file 3).

### Quality control of allele counting

Retrieving allele counts from RNA-seq data over a list of heterozygous sites is conceptually very simple, but several non-trivial filtering steps need to be undertaken to ensure that only high-quality reads representing independent RNA/cDNA molecules are counted. The first commonly applied filter is to remove reads with a potentially erroneous base over the heterozygous site based on low base quality. Furthermore, potential overlap of mates in paired-end RNA-seq data needs to be accounted for, so that each fragment, representing one RNA molecule, is counted only once per het-SNP. In the Geuvadis data, an average of 4.4 % of reads mapping to het-SNPs per sample are derived from overlapping mates, but this number will vary by the insert size (Figure S4a in Additional file 4).

In RNA-seq analysis, duplicate reads with identical start and end positions are common (15 % of reads in Geuvadis AE analysis), because highly expressed genes get saturated with reads (Figure S4b, d in Additional file 4). Thus, by default, duplicates are usually not removed from RNA-seq data to avoid underestimating expression levels in highly expressed genes [5]. However, we observe consistent albeit infrequent signs of PCR artifacts in the Geuvadis AE data, especially affecting lowly covered sites — where duplicates are mostly true PCR duplicates, since saturation is unlikely. Removing duplicate reads reduces technical sources of AE at these sites, while having a minimal effect on highly covered, read-saturated SNPs (Figure S4e in Additional file 4). Thus, we suggest that removing duplicate reads is a good default approach for AE analysis, and it is implemented as a default in the GATK tool. However, it is important that the retained read is either chosen randomly or by base quality, and not by mapping score, so as not to bias towards the reference allele.

The most difficult problem in AE analysis and a potential source of false positive AE is ensuring that 1) all the reads counted over a site indeed originate from that genomic locus, and 2) all reads from that locus are counted. RNA-seq studies with shorter or single-end RNA-seq reads are more susceptible to these problems. First, to make sure that no alien reads get erroneously assigned to a locus, only uniquely mapping reads should be used. This implies that highly homologous loci — such as microRNAs — are not amenable to AE analysis.

An even more difficult caveat in AE analysis is allelic mapping bias: in RNA-seq data aligned to the reference genome, a read carrying the alternative allele of a variant has at least one mismatch, and thus has a lower probability to align correctly than the reference reads [26–28]. Simulated data in Panousis et al. [27] indicates substantial variation between sites — in most cases reads mapped correctly, but 12 % of SNPs and 46 % of indels had allele ratio bias >5 % with some having a full loss of mapping of the alternative allele. Loci with homology elsewhere in the genome are particularly problematic as reads have nearly equally good alternative loci to align to. Furthermore, even a site with no bias in itself can become biased due to a flanking (sometimes unknown) variant that shares overlapping reads with the site of interest. In addition, mapping bias varies depending on the specific alignment software used (Additional file 5).

**Fig. 3:**
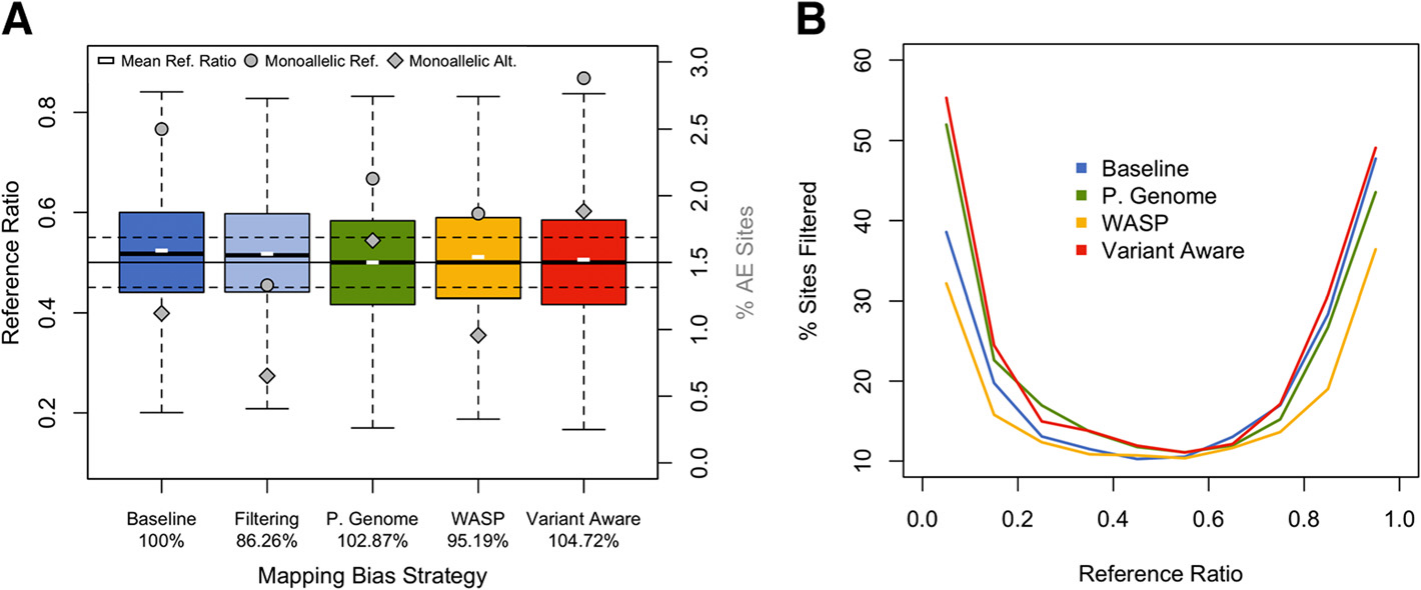
Strategies for reducing mapping bias in AE analysis. **a** Summary of various strategies to correct for mapping bias (*Baseline* = STAR aligned only, *Filtering* = STAR aligned with bias and mappability filters, *P. Genome* = STAR aligned to a personalized genome generated with Allele-Seq, *WASP* = STAR aligned with removal of biased reads using WASP, *Variant Aware* = GSNAP in variant aware alignment mode). The boxplot (axis on the left) shows reference ratios for AE sites covered by eight or more reads. The mean reference ratio for each strategy is shown with a *white dash*; the *solid black line* indicates a reference ratio of 0.5, while *dotted lines* indicate ±0.05. The percentages of sites that are monoallelic reference (*grey circle*) or alternative (*grey diamond*) are plotted against the secondary axis. The number of sites with AE data for each strategy is shown as a percentage of the baseline strategy underneath their respective labels. Outliers are hidden for ease of viewing. **b** Percentage of sites that are removed when bias and mappability filters are applied to resulting data from all strategies, shown for each reference ratio bin

Various strategies can be employed to control for the effect of mapping bias on AE analysis. The simplest approach that can be applied to AE data without realignment is to filter sites with likely bias [5, 8, 28]. In previous work [5, 8, 29–31] and in this paper, unless mentioned otherwise, we remove about 20 % of het-SNPs that either fall within regions of low mappability (ENCODE 50 bp mappability score < 1) or show mapping bias in simulations [27]. This reduces the number of sites with strong bias by about 50 % (Fig. 3b) but the genome-wide reference ratio remaining slightly above 0.5 indicates residual bias (Figure S6a in Additional file 6). Using this ratio as a null in statistical tests instead of 0.5 [5, 6] can improve results (Figure S6b–e in Additional file 6). More exhaustive but computationally intensive approaches include alignment to personalized genomes [18, 32, 33], or use of a variant-aware aligner, such as GSNAP [34]. These methods yield comparable results and eliminate *average* genome-wide bias (Fig. 3a; Additional file 5), but the fact that applying a mappability filter still removes monoallelic sites implies that not all bias is eliminated (Fig. 3b). In particular, in personalized or variant-aware approaches sites with homology elsewhere in the genome can have very substantial allelic mapping bias towards either the reference or non-reference allele, which occurs when reads carrying one allele map perfectly and reads with the other allele align to multiple loci. A novel approach is the specific removal of reads that show mapping bias with software such as WASP [35], which generally performs well, although some signs of residual bias still remain. Additional file 7 presents a summary of the strengths and weaknesses of each strategy. Altogether, while many approaches yield reasonably accurate data, allelic mapping bias remains a problem that cannot be perfectly eliminated with available solutions.

### Quality control of genotype data

AE analysis relies on data of heterozygous sites to distinguish the two parental alleles. These genotype data are ideally retrieved from DNA-sequencing or genotyping arrays, but the RNA-seq data themselves can also be used for calling genetic variants and finding heterozygous sites [36–39]. However, true allelic imbalance can lead to heterozygous sites being called homozygous in RNA-based genotype calling and lead to substantial error in monoallelic genes due to, e.g., imprinting, and more subtle bias in expression quantitative trait loci (eQTL) genes (Figure S7a in Additional file 8).

**Fig. 4:**
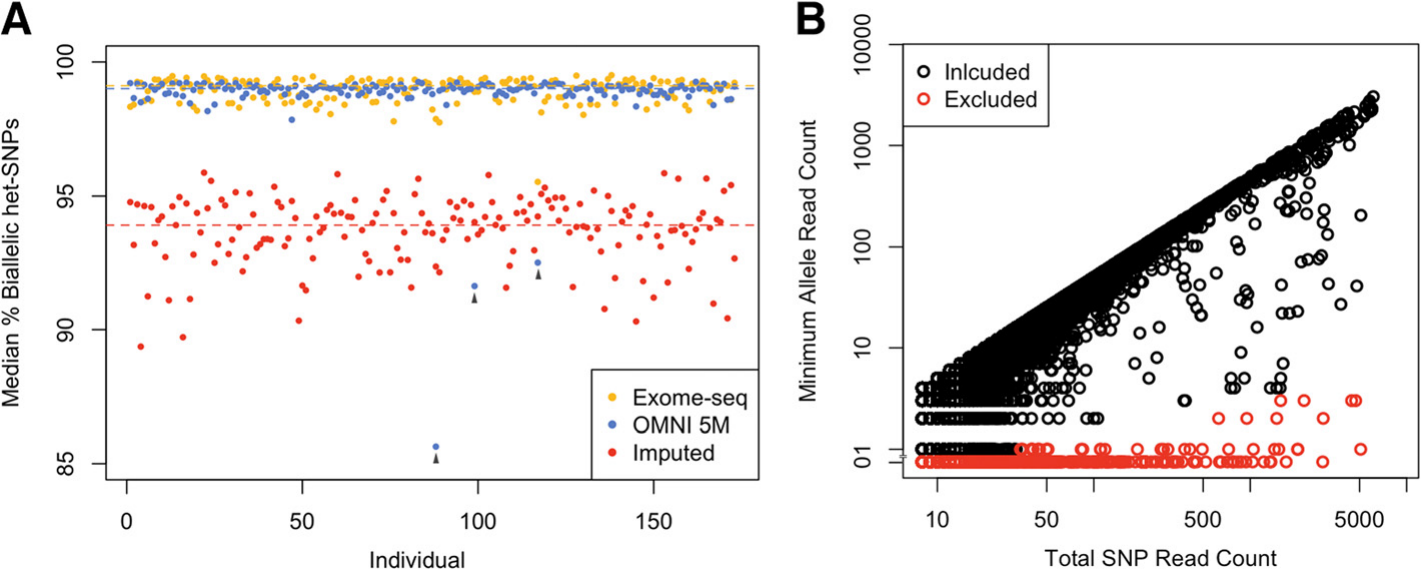
Quality control of genotype data for AE analysis. **a** Median percentage of het-SNPs where RNA-seq reads from both alleles are observed across all tissues for GTEx samples, genotyped with different platforms: exome-seq (*yellow*), Illumina OMNI 5 M SNP array (*blue*), and sites imputed from OMNI 5 M genotype array (*red*). *Grey arrowheads* indicate outlier individuals that are likely to have lower genotype quality. **b** Total het-SNP read count versus the read count of the lesser-covered allele for an individual Geuvadis sample. Sites flagged as putative genotyping errors are marked in *red*, with RNA-seq data not rendering support for heterozygosity

Even when using heterozygous genotypes called from DNA data, genotyping error can be an important source of false signals of allelic imbalance, because AE data from a homozygous site appear as monoallelically expressed. In genotype data that has passed normal quality control (QC), including Hardy-Weinberg equilibrium test, genotype error will lead to rare cases of monoallelic expression per site, not shared across many individuals (Fig. 1b). False heterozygous genotype calls are rare but not negligible in AE analysis using SNP genotypes from arrays or from modern sequencing data, but much more common in imputed data (Fig. 4a). Calculating the genome-wide proportion of monoallelic AE sites per individual is a sensitive method for genotyping quality control (Fig. 4a, arrowheads).

Removing genotyping error is relatively straightforward for analysis of moderate allelic imbalance (such as that caused by *cis*-regulatory variants): removing monoallelic variants removes sites with false genotypes and results in little loss of truly interesting data. However, highly covered sites are rarely strictly monoallelic even in a homozygous state due to rare errors in sequencing and alignment (Figure S7b in Additional file 8). Thus, we propose a genotype error filter where the average amount of such sequencing noise per sample is first estimated from alleles other than reference (REF) or alternative (ALT) (Figure S7c in Additional file 8). Then, binomial testing is used to estimate if the counts of REF/ALT alleles are significantly higher than this noise, and sites where homozygosity cannot be thus rejected are flagged as possible errors (Fig. 4b). Additionally, it may be desirable to flag fully monoallelic sites with low total counts, where homozygosity cannot be significantly rejected, but heterozygosity is not supported either. This test can also be applied to study designs with RNA-seq data from multiple samples (e.g., tissues or treatments) of a given individual, genotyped only once, since genotyping error causes consistent monoallelic expression in every tissue. In the Geuvadis data set with 1000 Genomes phase 1 genotypes and sites covered by eight or more reads, an average of 4.3 % of sites per sample are excluded by these criteria [1 % false discovery rate (FDR)].

Unfortunately, genotyping error is very difficult to distinguish from a true biological pattern of strong monoallelic expression, shared across all studied tissues, and present in a small number of samples, such as analysis of nonsense-mediated decay triggered by a rare variant, or a rare severe regulatory mutation (Fig. 1). The only real solution is rigorous genotype quality control and/or validation, and taking the possibility of confounding by genotyping error into account in interpretation of the results.

Sample mislabeling or mixing of the RNA-seq samples can lead to a substantial number false positive hits — as opposed to reduction of power in eQTL studies. Fortunately, simple metrics from AE analysis provide a sensitive way to detect sample contamination and mislabeling [40]. DNA-RNA heterozygous concordance — i.e., the proportion of DNA-heterozygous sites that are heterozygous also in RNA data — and a measure of allelic imbalance detect outliers and indicate the type of error (Figure S7d in Additional file 8).

### Technical covariates

**Fig. 5:**
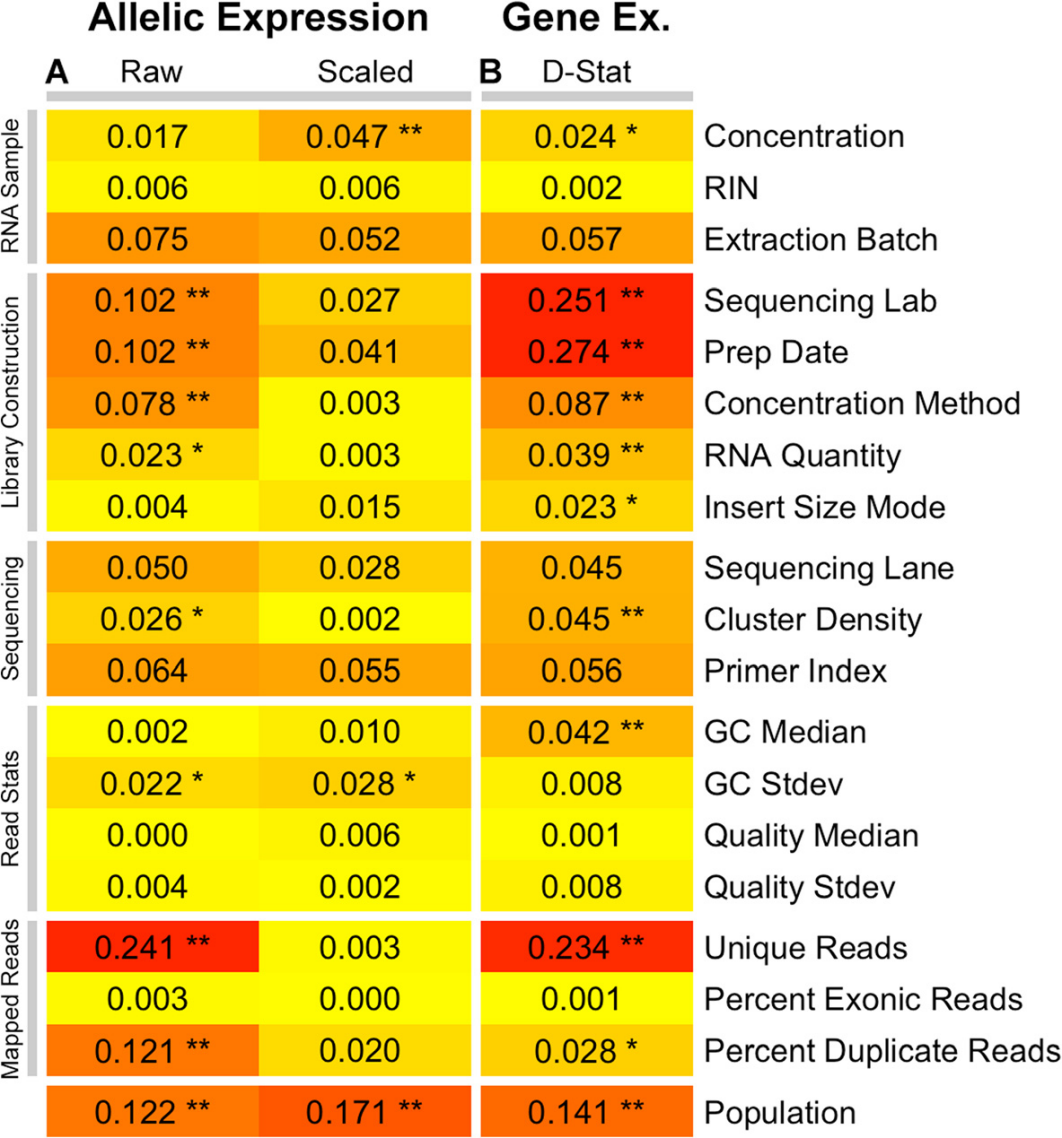
Technical covariates of AE. **a** Correlation of AE with technical covariates, measured as correlation (R^2^) between each covariate and the percentage of significant AE sites in a sample (binomial *p* < 0.05, het-SNPs with ≥30 reads), both before and after scaling to 30 reads. **b** Correlation of gene expression with technical covariates. As the gene expression statistic we use the median correlation of each sample to all other samples (D-statistic). Correlation to a biological covariate (population) is shown for comparison. Correlations were calculated from all Geuvadis samples by Spearman correlation for continuous covariates, or linear regression for categorical covariates. **p < 0.01, *p < 0.05, after Bonferroni correction. *RIN* RNA integrity number, *Stdev* standard deviation

RNA-seq has become a mature and highly reproducible technique, but it is not immune to technical covariates such as the laboratory which experiments were performed in, aspects of library construction and complexity, and sequencing metrics [40]. Gene expression studies are particularly susceptible to these technical factors, because read counts *between* samples are compared. AE analysis has the advantage that only read counts *within* samples are compared (allele versus allele), which makes it less susceptible to technical artifacts. We analyzed the correlation of the proportion of significant AE sites (binomial test, nominal *p* < 0.05) with various technical covariates in the Geuvadis data (Fig. 5a). In raw AE count data, we observe a high correlation with the library depth (unique reads; R^2^ = 0.24) — expectedly, since total read count of AE sites determines the statistical power to see significant effects (see below). In AE data corrected for variation in read counts by scaling the counts to 30, all technical correlations are very small and mostly non-significant, in stark contrast to gene expression level data that display strong batch effects (Fig. 5b). Thus, when appropriate measures are taken, AE analysis is an extremely robust approach that suffers less from technical factors than gene expression studies.

### Statistical tests for AE

**Fig. 6:**
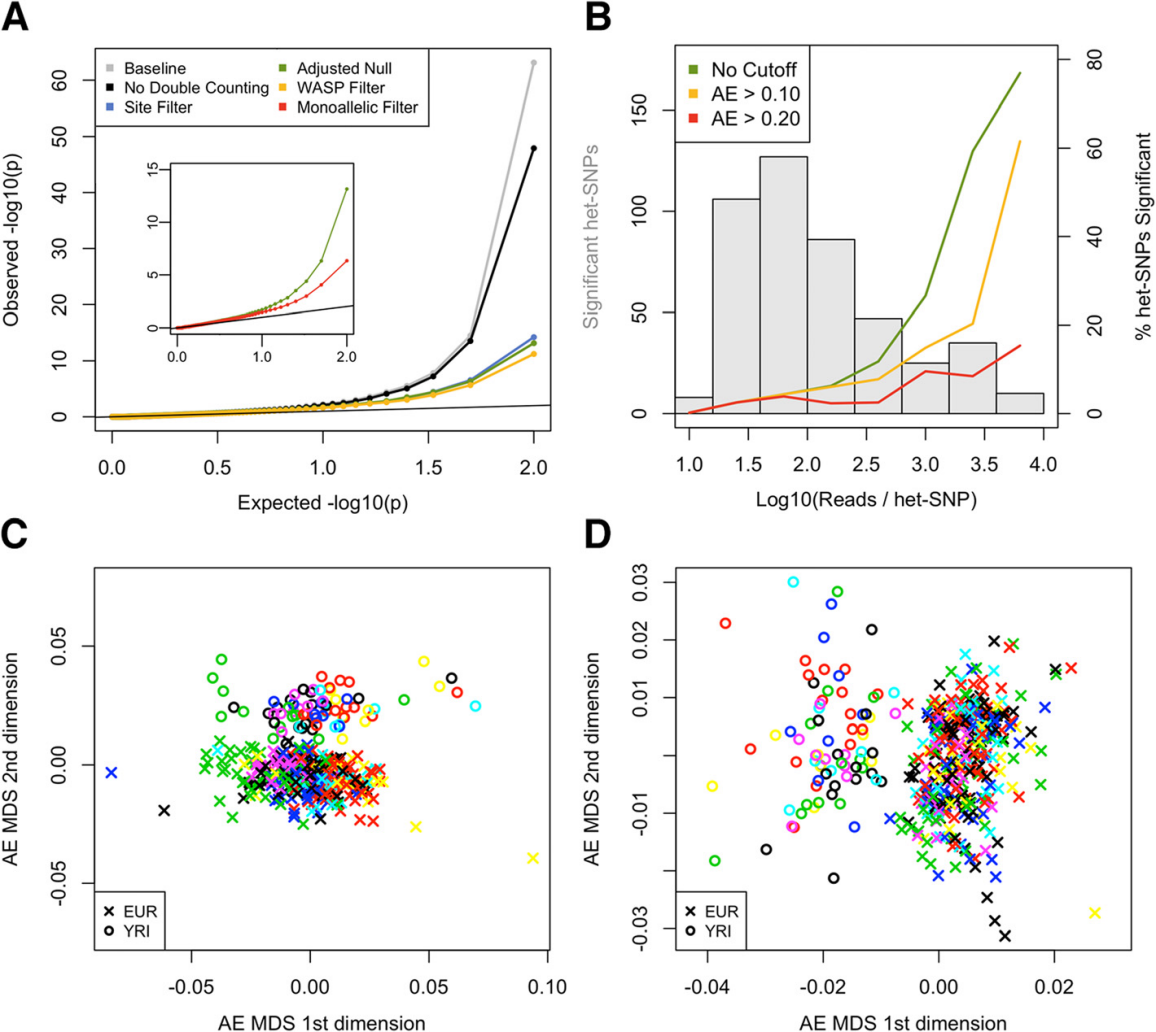
QC measures reduce false positives, demonstrated with a binomial test for allelic imbalance. **a** QQ plot of *p* values generated from binomial testing after various QC measures. *Baseline* = STAR aligned testing against a null of 0.5 without any correction for double counting, mapping bias, or genotyping error; *No Double Counting* = as Baseline but without duplicates and overlapping mate pairs counted once; *Site Filter* = as No Double Counting but without biased and low mappability het-SNPs; *Adjusted Null* = As Site Filter but using mean per base reference ratio as the binomial null; *WASP Filter* = as Site Filter but with WASP filtering of reads; *Monoallelic Filter* = as Adjusted Null but removing monoallelic sites to account for putative genotyping error. **b** Histogram showing distribution of coverage for sites with significant (5 % FDR) allelic imbalance according to a binomial test (primary axis), and the percentage of all het-SNPs that show significant allelic imbalance in each coverage bin using increasing allelic effect cutoffs (secondary axis). **c**, **d** Multidimensional scaling (*MDS*) clustering of Geuvadis samples based on proportion of sites with significant AE that differs between sample pairs. Samples are colored by sequencing laboratory and labeled by population. If significant sites are assigned based on a simple binomial test (FDR 5 %), the samples cluster first by sequencing laboratory due to lab-specific differences in coverage (**c**). This effect is mostly removed in (**d**) by requiring significant sites to have FDR 5 % and effect size > 0.15

A binomial test is the classic way to determine whether the ratio of the two alleles is significantly different from the expected 0.5, and has been widely used [2, 5, 8, 31]. However, AE data are overdispersed compared with what is expected under a binomial distribution, likely as a result of both biological and technical factors [35, 41, 42]. These technical factors arise from systematic artifacts such as allelic mapping bias, as well as from imperfect reproducibility (measurement error), which we were able to estimate using eight technical replicates of five Geuvadis samples [40]. Accounting for duplicates and overlapping read mates reduced measurement error between replicates (Additional file 9), with very low level of residual variation between replicates except for the highly covered sites (>500), although we note that this may not apply to all data sets. The other QC measures described above remove systematic artifacts and reduce the inflation of binomial *p* values further (Fig. 6a). Nonetheless, the binomial *p* values remain inflated, and especially highly covered sites are likely to have remaining systematic artifacts (Fig. 6b). This suggests that a simple binomial test may not be an appropriate statistical test for allelic imbalance because it could result in a high number of false positives. However, given that most genes have eQTLs [4, 5, 8], biological sources of AE are expected to be extremely widespread, which is further supported by high heritability of AE [2]. Thus, while various statistical models have been put forward, many of which use variations of a beta-binomial model to infer the level of overdispersion [35, 41, 42], it remains inherently difficult to distinguish biological sources of overdispersion from putative technical effects. One approach is to analyze AE across individuals and tissues to control for confounders and capture the biological signal of interest — such as *cis*-regulatory variation [35, 41], imprinting [13], or nonsense-mediated decay [20]. However, many of the statistical approaches to analyze AE data are just emerging, and their full benchmarking is beyond the scope of this paper. For reference, a list of the currently available tools and publications that analyze AE data, including their specific biological application, statistical test used, and required inputs, can be found in Additional file 10.

Often during AE analysis the intent is to compare allelic imbalance between different sites, or between individuals. This is complicated by the highly variable total read counts at het-SNPs (Fig. 2a), since they lead to substantial differences in statistical power at different sites. These differences are driven by differences in library depth between samples, as well as biologically variable expression levels between genes and samples. Such differences can cause samples to cluster by experimental batch (Fig. 6c). If the goal of the analysis is to capture AE, patterns introduced by expression levels are often not desirable. While this problem ultimately needs to be addressed with tailored statistical approaches, it can be alleviated with a straightforward minimum effect size cutoff that reduces the enrichment of significant sites in highly covered het-SNPs (Fig. 6b), and accounts for the strongest dependency of total read counts (Fig. 6d). An experimental approach is to use an assay that yields high read counts, such as mmPCR-seq, instead of or alongside RNA-seq data [9, 12, 13, 43].

### QC measures improve the power to detect biologically relevant AE

**Fig. 7:**
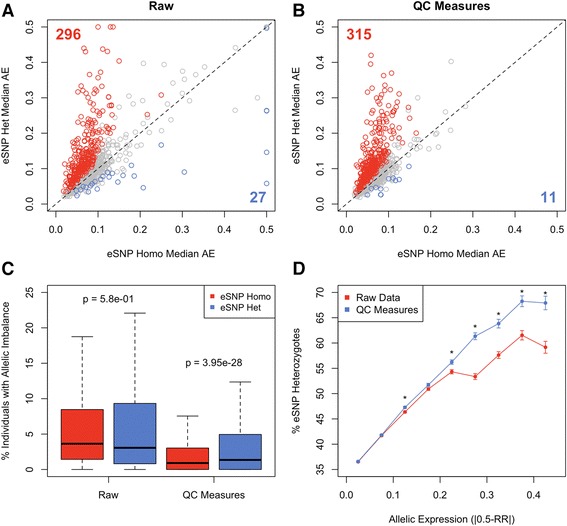
QC measures improve the power to detect biologically relevant AE at genes that have eQTLs (eGenes), where individuals that are heterozygous for the top eQTL SNP (eSNP) are expected to have more AE than homozygous individuals. Plot of median AE in heterozygous versus homozygous individuals for each eGene, before (**a**) and after (**b**) QC measures. *Red points* indicate a significant (1 % FDR) difference in AE level in the expected direction (AE het > AE homo, true positive), *blue points* indicate a significant difference in the opposite direction (AE het < AE homo, false positive), and the number of true and false positives is listed. **c** Boxplot of the percentage of individuals showing allelic imbalance (AE > 0.25) who are either heterozygous or homozygous for the top eQTL at each eGene before and after QC measures. Outliers are hidden for ease of viewing. **d** Mean percentage of het-SNPs that are found within heterozygous eGenes in bins of AE across individuals before and after QC measures. *Error bars* represent the standard error of the mean, and *asterisks* indicate a significant difference (1 % FDR) after applying QC measures for that bin

Regardless of the specific application, the QC measures proposed here should increase true signals of AE, resulting in improved power to detect biological phenomena of interest. To demonstrate this, we analyzed AE at 1154 genes with known eQTL (eGenes) in 343 European individuals using Geuvadis LCL RNA-seq data [5]. Individuals who are heterozygous for an eQTL SNP (eSNP) are expected to show increased AE within the eGene compared with those who are homozygous. Applying QC measures increased the significance of the difference in AE and reduced the variance of AE at eGenes (Additional file 11). Altogether this increased the power to distinguish between AE levels in eSNP heterozygous versus homozygous eGenes, with a 6.8 % increase in true positives, and 59.3 % decrease in false positives after applying QC measures (Fig. 7a, b). The measures also significantly increased the difference in the proportion of individuals exhibiting allelic imbalance (AE > 0.25) between the two classes (Fig. 7c), and resulted in a robust enrichment of sites within heterozygous eQTL across the spectrum of allelic imbalance (Fig. 7d). These results clearly illustrate the immediate benefit of ensuring AE data used for analysis are of high quality by applying the QC measures outlined here.

## Conclusion

In this paper, we have introduced tools for retrieving high-quality AE data from RNA-seq data sets. We have described how the quality of the input data affects AE analysis, and outlined the QC approaches that are needed to obtain accurate estimates of AE from RNA-seq data (Additional files12 and 13). Altogether, we show that carefully collected and filtered AE estimates from modern RNA-seq data are remarkably robust to technical variation in RNA-seq data, highlighting their utility for detecting diverse biological phenomena of genetic and epigenetic variation. Increasingly standardized production of AE data advances wider data sharing and integration across studies, although the genotype data included in AE estimates by default pose limitations on data access. The increasing amounts of AE data from large-scale RNA-seq studies hold great promise for capturing regulatory variation even in small numbers of samples, allowing integrated analysis of the personalized genome and its function.

## Materials and methods

### GATK ASEReadCounter tool and benchmarking

The tool and accompanying documentation are available in GATK v.3.4, which can be downloaded from [44]. The Python script which processes the output from SAMtools mpileup can be found at [45]. Benchmarking was run using GATK v.3.4 and SAMtools 1.2 on STAR aligned reads from the Geuvadis sample NA06986.2.M_111215_4 using heterozygous bi-allelic sites from 1000 Genomes phase 1. Reads were coordinate sorted, indexed, and WASP filtered to produce a BAM file containing 56,362,192 reads. Runtime benchmarking was performed using 100 %, 75 %, and 50 % of the reads sampled from the file, and is reported as the mean of 10 runs with the 95 % confidence interval shown. For comparison ASEQ v.1.1.8 was run in pileup mode. Benchmarking was run on CentOS 6.5 with Java version 1.6 on an Intel Xeon CPU E7- 8830 @ 2.13GHz.

### Filtering homozygous sites

In order to identify potentially homozygous sites miscalled as a heterozygous SNP we model the number of reads that can be observed due to technical error of the experimental and upstream computational pipeline. Let us assume there are a total of *n* reads originating from a site homozygous for an allele R. Assuming a noise rate *ε*, by which a read can erroneously support another allele A, the distribution of total number of reads aligned to allele A, *n*_*A*_, is given by binomial distribution. Hence, the probability of observing *n*_*A*_ or more reads assigned to allele A in a site homozygous for R is given by:

$$ p\left(x\ge {n}_A\Big|\ RR\right)=1- BinCDF\left({n}_A,n,\varepsilon \right), $$

where *BinCDF*(*n*_*A*_, *n*, *ε*) is the binomial cumulative distribution function. Conversely, the probability of observing *n*_*R*_(*n* = *n*_*R*_ + *n*_*A*_) or more reads assigned to allele R in a site homozygous for A is given by:

$$ p\left(x\ge {n}_R\Big|\  AA\right)=1- BinCDF\left({n}_R,n,\varepsilon \right), $$

under the assumption that the noise rate is equal for all alleles. Therefore, the probability of observing extreme allelic imbalance due to the null hypothesis, homozygosity for one of the alleles, can be calculated by summing up the two above probabilities corresponding to the two tails of the distribution. In order to derive an empirical estimate of the noise rate *ε* we used the ratio between the total sum of reads assigned to other alleles, those different from the designated reference or alternative allele at each site, to the total number of reads in a library divided by two. For this purpose we exclude the sites with more than 5 % of the reads aligned to other alleles from the analysis.

### Mapping strategies for AE analysis

For all analyses, unless otherwise noted, reads were mapped using STAR v.2.4.0f1 and the two-pass mapping strategy as recommended by the Broad Institute [39]. Briefly, splice junctions are detected during a first pass mapping, and these are used to inform a second round of mapping. All reads were mapped to hg19 and Gencode v19 annotations were used.

For mapping to a personalized genome, the vcf2diploid tool, part of AlleleSeq, was used to generate both a maternal and paternal genome for NA06986 from the phased 1000 Genomes phase 1 reference using het-SNPs only. Reads were then mapped to both genomes separately using STAR two-pass strategy (as above). Reads which aligned uniquely to only one genome were kept, and in cases where reads mapped uniquely to both genomes, the alignment with the higher alignment quality was used.

Mapping using GSNAP was performed with default settings and splice site annotations from hg19 refGene. Variant-aware alignment was performed using the “-d” option for NA06986 from the phased 1000 Genomes phase 1 reference using het-SNPs only, as described in the GSNAP documentation.

### Multidimensional scaling clustering of samples by AE data

A pairwise distance matrix was produced for all Geuvadis samples using AE data and used for classical multidimensional scaling (cmdscale) in R. The first two dimensions were then plotted against each other for all samples. The distance between two samples was calculated as follows: Pairwise distance = Total number of sites with significant AE in only one sample/Total number of shared sites. A binomial test with a 5 % FDR was used for significance with either no effect size cutoff (Fig. 6c) or a minimum effect size of 0.15 (Fig. 6d).

### Measuring AE at eQTL genes

RNA-seq data from 343 Geuvadis European individuals was used to generate allele counts at het-SNPs. For each individual, AE (AE = |0.5 − Reference ratio |) was calculated for all sites with ≥16 reads, each site was intersected against all Geuvadis European genes with a significant eQTL (eGene, 5 % FDR), and the median AE of all sites covering each eGene was calculated. The genotype of each individual for the top eQTL for each gene was then determined to be either heterozygous or homozygous. For each eGene with at least 30 measurements of AE in both heterozygous and homozygous individuals the significance of the difference in AE between the two classes was calculated using a Wilcoxon rank sum test (1 % FDR). To determine the enrichment of sites within eSNP heterozygous eGenes across the AE spectrum, the percentage of these sites was calculated in bins of AE for each individual.

### Units of AE

For a single variant:

- Reference ratio = Reference reads/Total reads
- Allelic expression (effect size) = |0.5 – Reference ratio|

### Data availability

RNA-seq data from the Geuvadis Consortium alongside 1000 Genomes phase 1 genotype data were used for all analyses. RNA-Seq FASTQ files are available from the European Nucleotide Archive under accession [ENA:ERP001942].

## Additional files

Additional file 1: Figure S1. Allelic expression signal from a population of monoclonal versus polyclonal cells. In the latter, standard RNA-sequencing will show allelic imbalance only when the two alleles are systematically differentially expressed, e.g., due to a regulatory variant or imprinting. (TIFF 3238 kb) Additional file 2: Figure S2. Genomic coverage of allelic expression data in Geuvadis CEU samples (extended). **a** Total number of unique het-SNPs covered by increasing read depth as a function of the number of individuals. **b** Boxplot of the total number of exons per individual containing at least one het-SNP for each depth level. **c** Median number of exons as a function of the number of het-SNPs per feature at increasing read depths. **d** Distribution of percentage of reads mapping to het-SNPs that cover more than one het-SNP for all Geuvadis samples (median = 8.8 %). (TIFF 1735 kb) Additional file 3: Figure S3. Performance of GATK ASEReadCounter (GATK) tool compared with SAMtools mpileup with output processed by a custom Python script. **a** Mean runtime in minutes to produce allele counts from a processed BAM file with 100 %, 75 %, and 50 % of the reads sampled (see "Materials and methods"). ASEQ running in pileup mode is included as a comparison. *Error bars* show a 95 % confidence interval generated from ten runs. Plot (**b**) and distribution (**c**) of reference ratios for sites covered by ≥30 reads calculated using read counts generated using either the GATK or SAMtools mpileup. (TIFF 4277 kb) Additional file 4: Figure S4. Effect of overlapping and duplicate reads on AE analysis of Geuvadis samples. **a** Histogram of percent overlapping mates of paired-end reads at het-SNPs used for AE analysis. **b** Histogram of percentage of duplicate reads at het-SNPs used for AE analysis. **c** Total coverage versus percentage of duplicate reads at AE sites. **d** Percentage of duplicate reads in coverage level bins for Geuvadis samples with the minimum (77.5 %, *red*), median (83.9 %, *yellow*) and maximum (89.6 %, *green*) read complexity at het-SNPs. Complexity is defined as Total number of reads mapping to het-SNPs after removing duplicates/Number of reads before removing duplicates. **e** Effect of duplicate removal on allelic expression effect size [AE = |0.5 – Reference reads/Total reads|, ∆AE = AE(Duplicates removed) – AE(No duplicates removed)] on het-SNPs binned by coverage level, sites where ∆AE = 0 are not shown. (TIFF 2407 kb) Additional file 5: Figure S5. Comparison of AE data generated with different alignment strategies. **a–d** For each comparison the observed reference ratios for het-SNPs that have AE data in both strategies are plotted against each other (*Shared het-SNPs*), histograms show the reference ratios of sites that are unique to only one analysis (*Unique het-SNPs*), and a density plot shows the genome wide reference ratio distribution for each analysis. *AS* = personalized genome generated with Allele-Seq and phased genotype data, *GSNAP vAWARE* = GSNAP using variant aware alignment. No filtering of sites has been done. All data come from Geuvadis LCL RNA-seq libraries from NA06986. Only het-SNPs with eight or more reads are included. (TIFF 15560 kb) Additional file 6: Figure S6. Low-level reference bias at het-SNPs remains after filtering biased sites. **a** Boxplot of reference ratio (Reference/Total) for each reference-alternative base combination for each Geuvadis sample, mapped with STAR two-pass and filtered for sites with low mappability or mapping bias in simulations as well as sites with potential genotyping error as described before. Ratio is calculated by summing up all REF and ALT read counts for that combination in a sample at sites that have eight or more reads, and for sites with coverage > 75^th^ percentile total counts were scaled down to the 75^th^ percentile to avoid sites with very high coverage having a disproportionate effect on the overall ratio. **b, c** Binomial test of AE on an example Geuvadis sample using an expected reference ratio of 0.5 (**b**) or against the calculated mean scaled reference ratio (**c**) (as described above), with sites of significant AE shown in *red* (5 % FDR). **d** Histogram of reference ratios at significant sites from (**b**). **e** Histogram of reference ratios at significant sites from (**c**). (TIFF 7345 kb) Additional file 7: Table S1. Summary of methods for correcting mapping bias in AE analysis. (XLSX 34 kb) Additional file 8: Figure S7. Quality control of genotype data for allelic expression analysis (extended). **a** Boxplot of per individual percentage of false homozygous RNA-seq genotype calls at het-SNPs in genes with *cis*-eQTLs in LCLs (FDR ≤ 0.05, Geuvadis), imprinted genes (based on [13] excluding genes detected exclusively in Geuvadis data), and all other genes. False homozygosity is defined as sites where variant calling using LCL RNA-seq data indicate the individual is homozygous for a non-reference allele, while DNA genotyping (1000 Genomes) indicates they are heterozygous. Genotype calls were made using GATK and best practices for RNA-seq genotype calling. **b** Percentage of het-SNPs where reads from foreign alleles (≥1 *blue*, ≥2 *green*, ≥3 *yellow*, ≥4 *red*) are observed as a function of coverage level using all Geuvadis RNA-seq data. Binned by hundreds of reads/het-SNP. **c** Frequency of the proportion of reads from foreign alleles (non-reference or alternative) observed (*ε*) in all Geuvadis samples (median = 4.128 × 10^-4^). **d** Scatterplot of percentage of significant AE sites (binomial test, *p* < 0.05) and percentage of biallelic het-SNPs (one or more read for each allele), for five Geuvadis libraries that have been contaminated with another sample in silico (0–75 % contamination). (TIFF 2131 kb) Additional file 9: Figure S8. QC measures reduce overdispersion in technical replicates when testing for allelic imbalance using a binomial test. Variance of allelic ratios as a function of total read counts, calculated as the mean at a given SNP from a Geuvadis individual with eight technical replicates (*grey*) with (**b**) or without (**a**) accounting for duplicate reads and overlapping read mates. The *lines* denote locally weighted smoothing of observed data (*black*) and theoretical variance for binomially distributed data (*red*). (TIFF 3209 kb) Additional file 10: Table S2. Summary of publications and tools that analyze AE data, listing their specific application, the type of statistical test performed, and the required input data. (XLSX 27 kb) Additional file 11: Figure S9. QC measures improve the power to detect biologically relevant allelic expression at genes that have eQTLs (eGenes), where individuals that are heterozygous for the top eQTL SNP (eSNP) are expected to have more allelic expression than homozygous individuals (extended). **a** QC measures increase the significance of the difference between heterozygous and homozygous individuals within eGenes. **b** QC measures reduce the variance of allelic expression between individuals within eGenes. (TIFF 2856 kb) Additional file 12: Figure S10. Complete workflow for AE analysis illustrating appropriate quality control measures and filters. (TIFF 782 kb) Additional file 13: Table S3. Summary of QC problems for AE data, proposed solutions, and potential drawbacks. (XLSX 31 kb)

## Abbreviations

AE: allelic expression
AS: allelic splicing
eQTL: expression quantitative trait locus
eSNP: expression quantitative trait locus single-nucleotide polymorphism
FDR: false discovery rate
GATK: Genome Analyzer Toolkit
het-SNP: heterozygous single-nucleotide polymorphism
indel: insertion or deletion
LCL: lymphoblastoid cell line
PCR: polymerase chain reaction
QC: quality control
RNA-seq: RNA-sequencing
SNP: single-nucleotide polymorphism
