# Supplementary figures and images for: Tools and best practices for data processing in allelic expression analysis
Source: Genome Biol. 2015 Sep 17;16(1):195. doi: 10.1186/s13059-015-0762-6 (PMC4574606; doi:10.1186/s13059-015-0762-6)

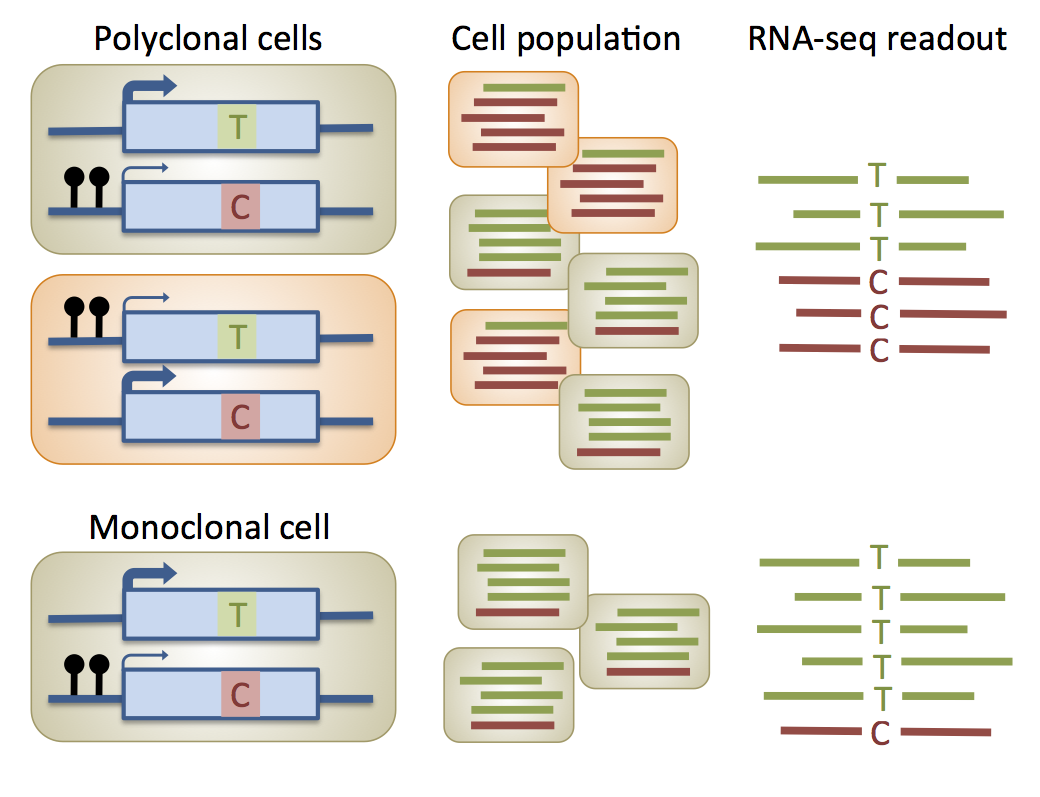

Supplement: Additional file 1: Figure S1. — Allelic expression signal from a population of monoclonal versus polyclonal cells. In the latter, standard RNA-sequencing will show allelic imbalance only when the two alleles are systematically differentially expressed, e.g., due to a regulatory variant or imprinting. (TIFF 3238 kb) [file 13059_2015_762_MOESM1_ESM.tif]

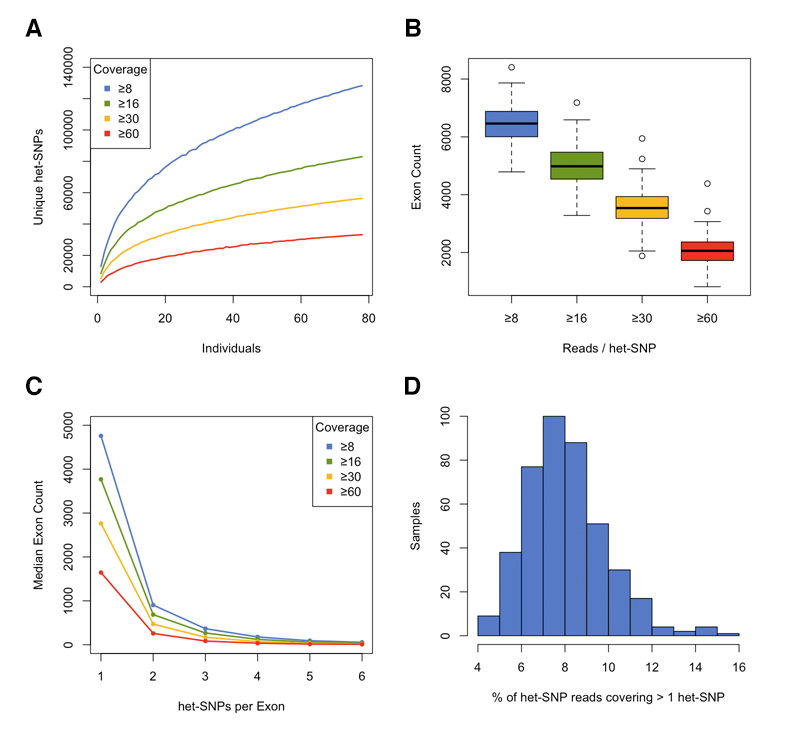

Supplement: Additional file 2: Figure S2. — Genomic coverage of allelic expression data in Geuvadis CEU samples (extended). a Total number of unique het-SNPs covered by increasing read depth as a function of the number of individuals. b Boxplot of the total number of exons per individual containing at least one het-SNP for each depth level. c Median number of exons as a function of the number of het-SNPs per feature at increasing read depths. d Distribution of percentage of reads mapping to het-SNPs that cover more than one het-SNP for all Geuvadis samples (median = 8.8 %). (TIFF 1735 kb) [file 13059_2015_762_MOESM2_ESM.tif]

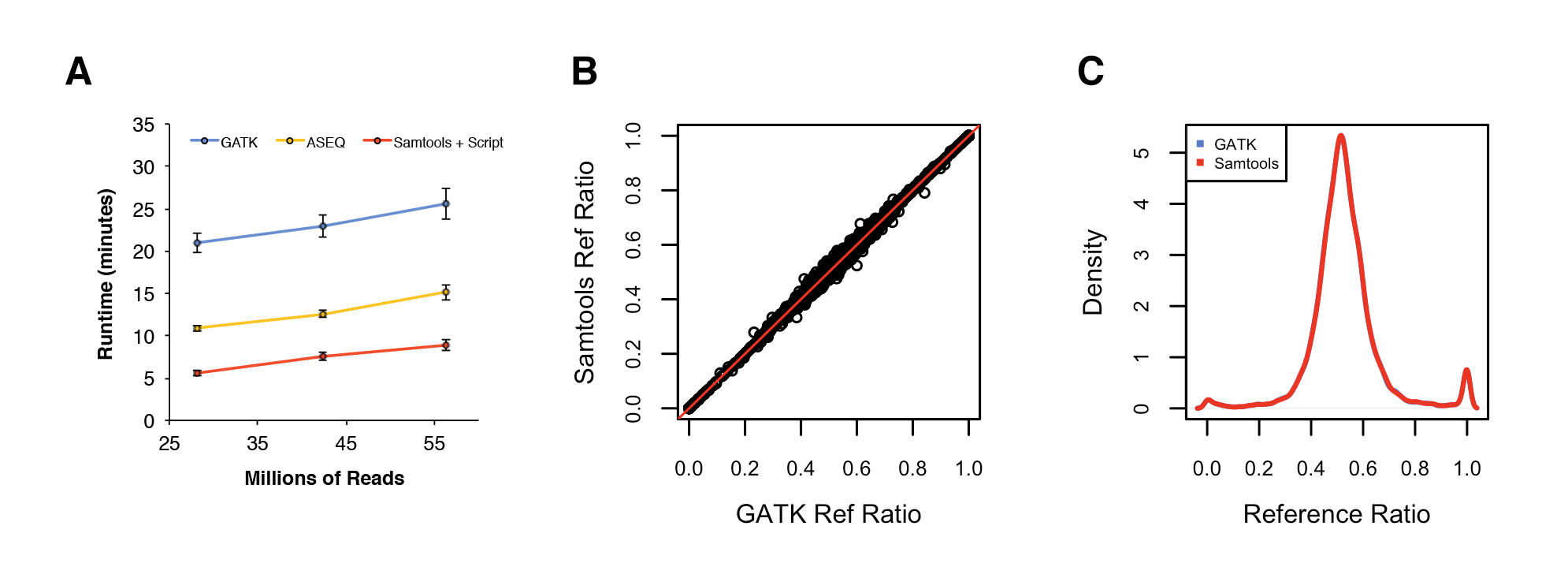

Supplement: Additional file 3: Figure S3. — Performance of GATK ASEReadCounter (GATK) tool compared with SAMtools mpileup with output processed by a custom Python script. a Mean runtime in minutes to produce allele counts from a processed BAM file with 100 %, 75 %, and 50 % of the reads sampled (see "Materials and methods"). ASEQ running in pileup mode is included as a comparison. Error bars show a 95 % confidence interval generated from ten runs. Plot (b) and distribution (c) of reference ratios for sites covered by ≥30 reads calculated using read counts generated using either the GATK or SAMtools mpileup. (TIFF 4277 kb) [file 13059_2015_762_MOESM3_ESM.tif]

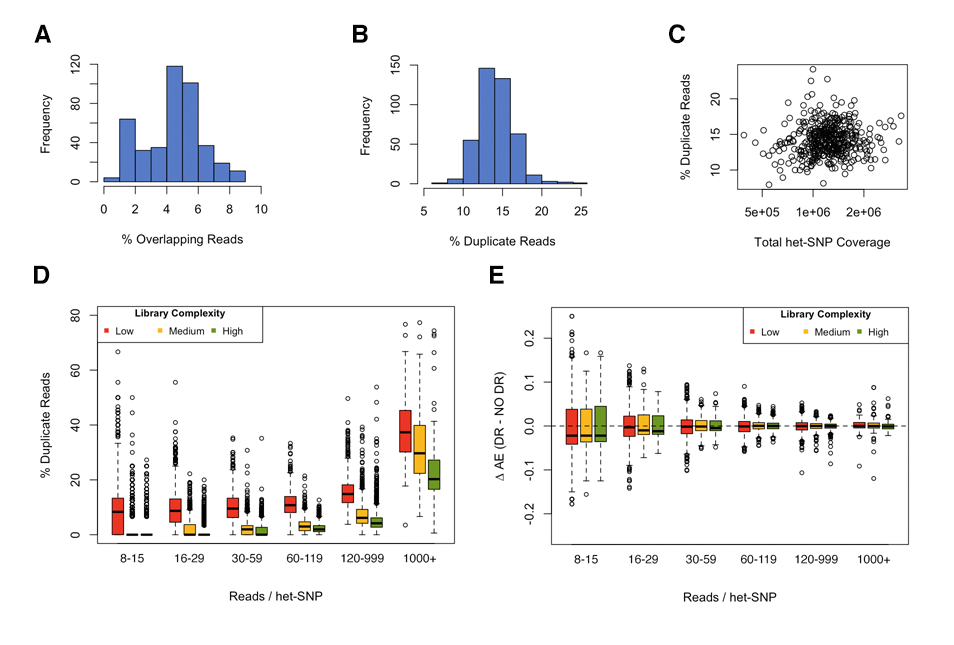

Supplement: Additional file 4: Figure S4. — Effect of overlapping and duplicate reads on AE analysis of Geuvadis samples. a Histogram of percent overlapping mates of paired-end reads at het-SNPs used for AE analysis. b Histogram of percentage of duplicate reads at het-SNPs used for AE analysis. c Total coverage versus percentage of duplicate reads at AE sites. d Percentage of duplicate reads in coverage level bins for Geuvadis samples with the minimum (77.5 %, red), median (83.9 %, yellow) and maximum (89.6 %, green) read complexity at het-SNPs. Complexity is defined as Total number of reads mapping to het-SNPs after removing duplicates/Number of reads before removing duplicates. e Effect of duplicate removal on allelic expression effect size [AE = |0.5 – Reference reads/Total reads|, ∆AE = AE(Duplicates removed) – AE(No duplicates removed)] on het-SNPs binned by coverage level, sites where ∆AE = 0 are not shown. (TIFF 2407 kb) [file 13059_2015_762_MOESM4_ESM.tif]

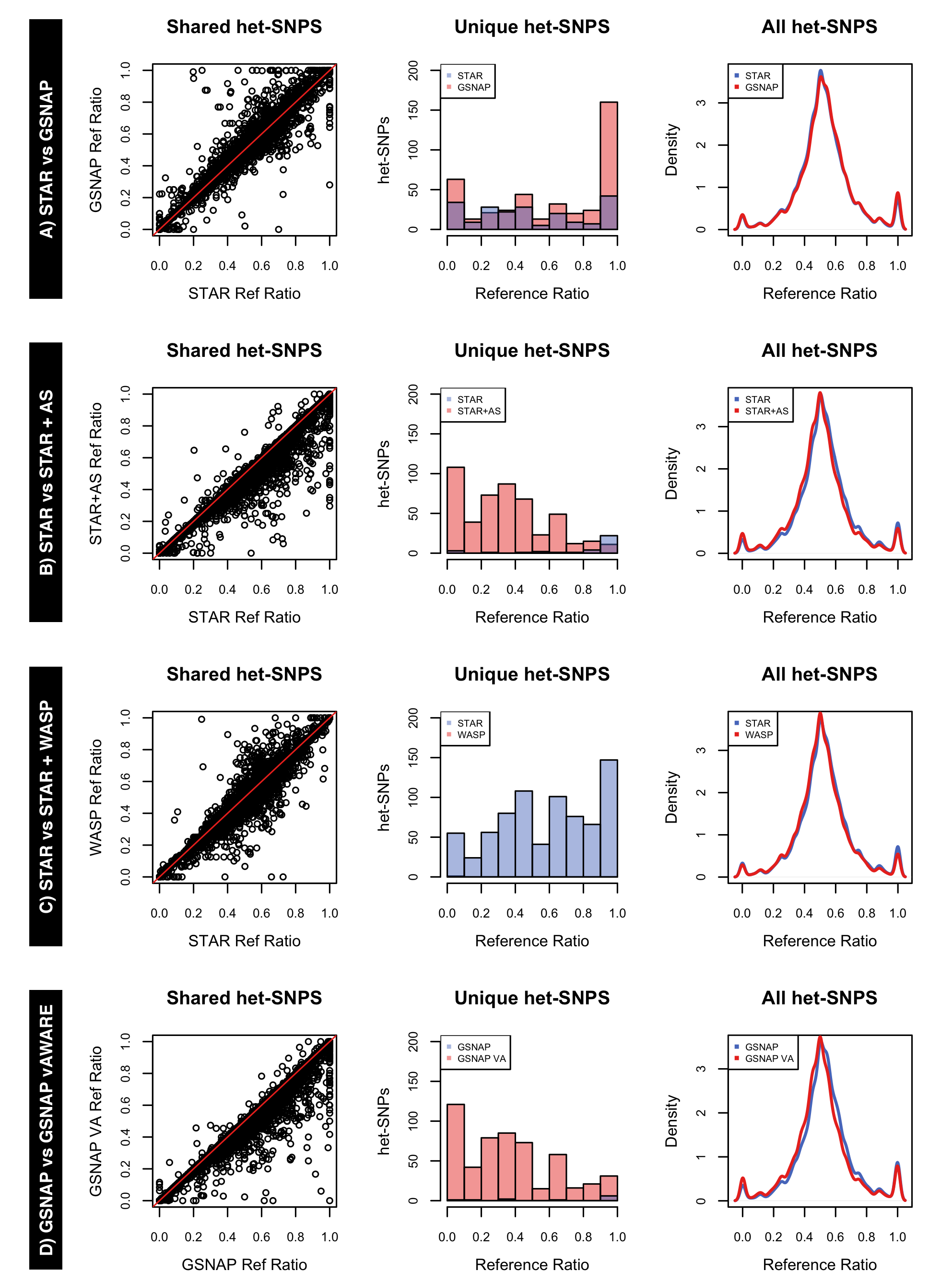

Supplement: Additional file 5: Figure S5. — Comparison of AE data generated with different alignment strategies. a–d For each comparison the observed reference ratios for het-SNPs that have AE data in both strategies are plotted against each other (Shared het-SNPs), histograms show the reference ratios of sites that are unique to only one analysis (Unique het-SNPs), and a density plot shows the genome wide reference ratio distribution for each analysis. AS = personalized genome generated with Allele-Seq and phased genotype data, GSNAP vAWARE = GSNAP using variant aware alignment. No filtering of sites has been done. All data come from Geuvadis LCL RNA-seq libraries from NA06986. Only het-SNPs with eight or more reads are included. (TIFF 15560 kb) [file 13059_2015_762_MOESM5_ESM.tif]

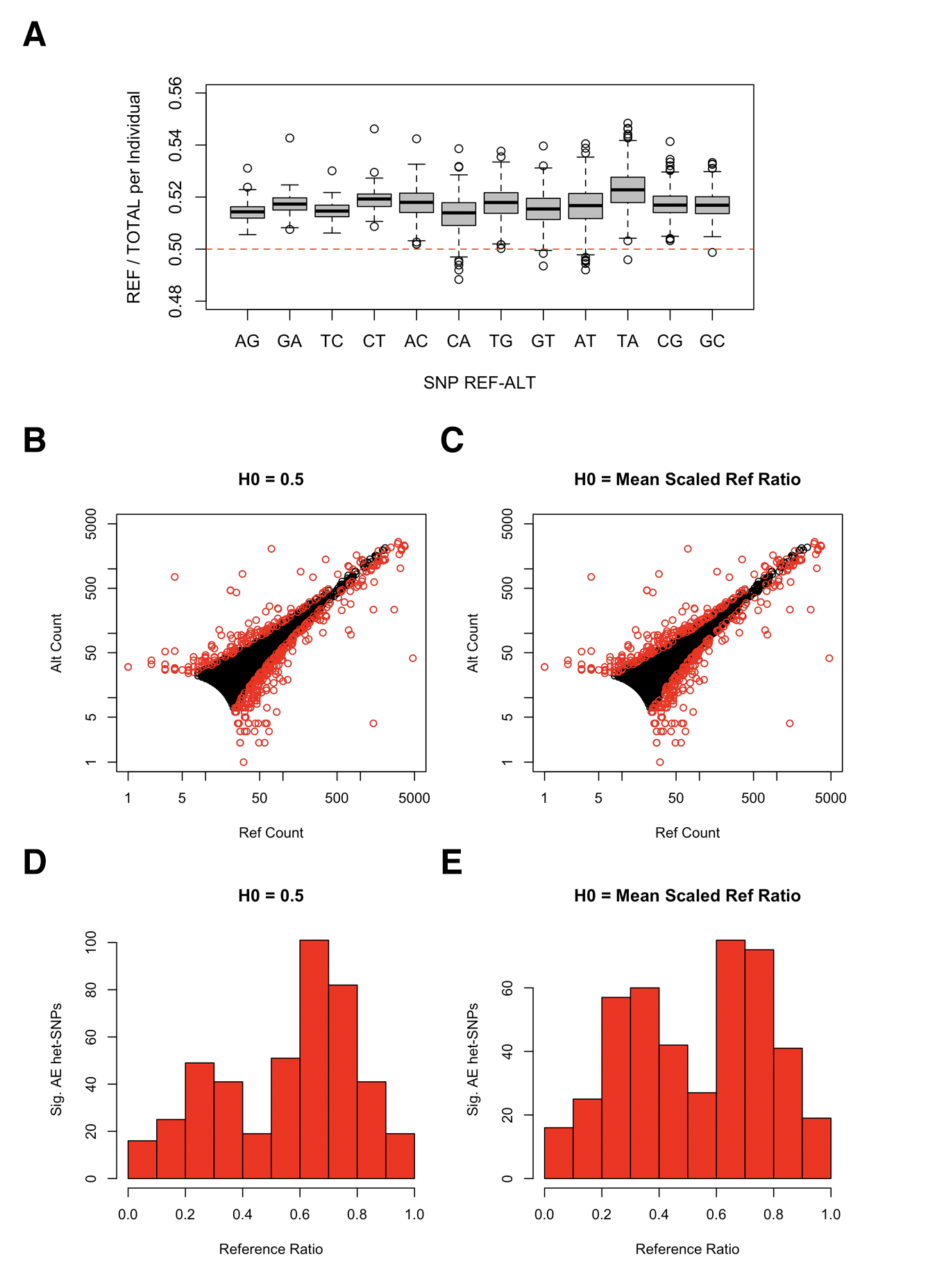

Supplement: Additional file 6: Figure S6. — Low-level reference bias at het-SNPs remains after filtering biased sites. a Boxplot of reference ratio (Reference/Total) for each reference-alternative base combination for each Geuvadis sample, mapped with STAR two-pass and filtered for sites with low mappability or mapping bias in simulations as well as sites with potential genotyping error as described before. Ratio is calculated by summing up all REF and ALT read counts for that combination in a sample at sites that have eight or more reads, and for sites with coverage > 75th percentile total counts were scaled down to the 75th percentile to avoid sites with very high coverage having a disproportionate effect on the overall ratio. b, c Binomial test of AE on an example Geuvadis sample using an expected reference ratio of 0.5 (b) or against the calculated mean scaled reference ratio (c) (as described above), with sites of significant AE shown in red (5 % FDR). d Histogram of reference ratios at significant sites from (b). e Histogram of reference ratios at significant sites from (c). (TIFF 7345 kb) [file 13059_2015_762_MOESM6_ESM.tif]

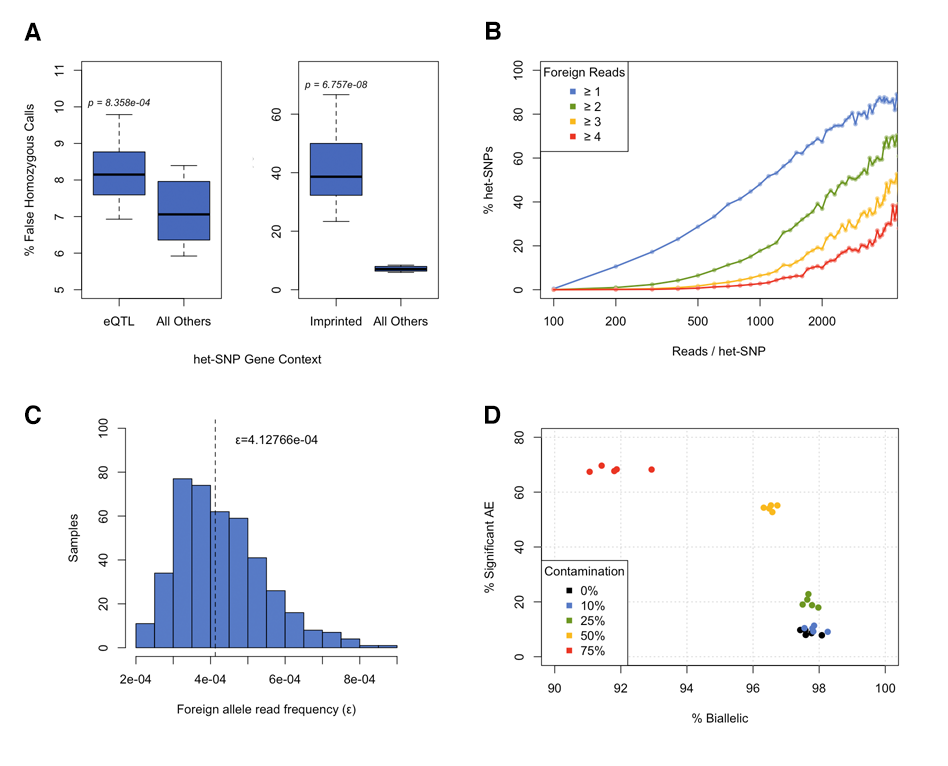

Supplement: Additional file 8: Figure S7. — Quality control of genotype data for allelic expression analysis (extended). a Boxplot of per individual percentage of false homozygous RNA-seq genotype calls at het-SNPs in genes with cis-eQTLs in LCLs (FDR ≤ 0.05, Geuvadis), imprinted genes (based on [13] excluding genes detected exclusively in Geuvadis data), and all other genes. False homozygosity is defined as sites where variant calling using LCL RNA-seq data indicate the individual is homozygous for a non-reference allele, while DNA genotyping (1000 Genomes) indicates they are heterozygous. Genotype calls were made using GATK and best practices for RNA-seq genotype calling. b Percentage of het-SNPs where reads from foreign alleles (≥1 blue, ≥2 green, ≥3 yellow, ≥4 red) are observed as a function of coverage level using all Geuvadis RNA-seq data. Binned by hundreds of reads/het-SNP. c Frequency of the proportion of reads from foreign alleles (non-reference or alternative) observed (ε) in all Geuvadis samples (median = 4.128 × 10^-4^). d Scatterplot of percentage of significant AE sites (binomial test, p < 0.05) and percentage of biallelic het-SNPs (one or more read for each allele), for five Geuvadis libraries that have been contaminated with another sample in silico (0–75 % contamination). (TIFF 2131 kb) [file 13059_2015_762_MOESM8_ESM.tif]

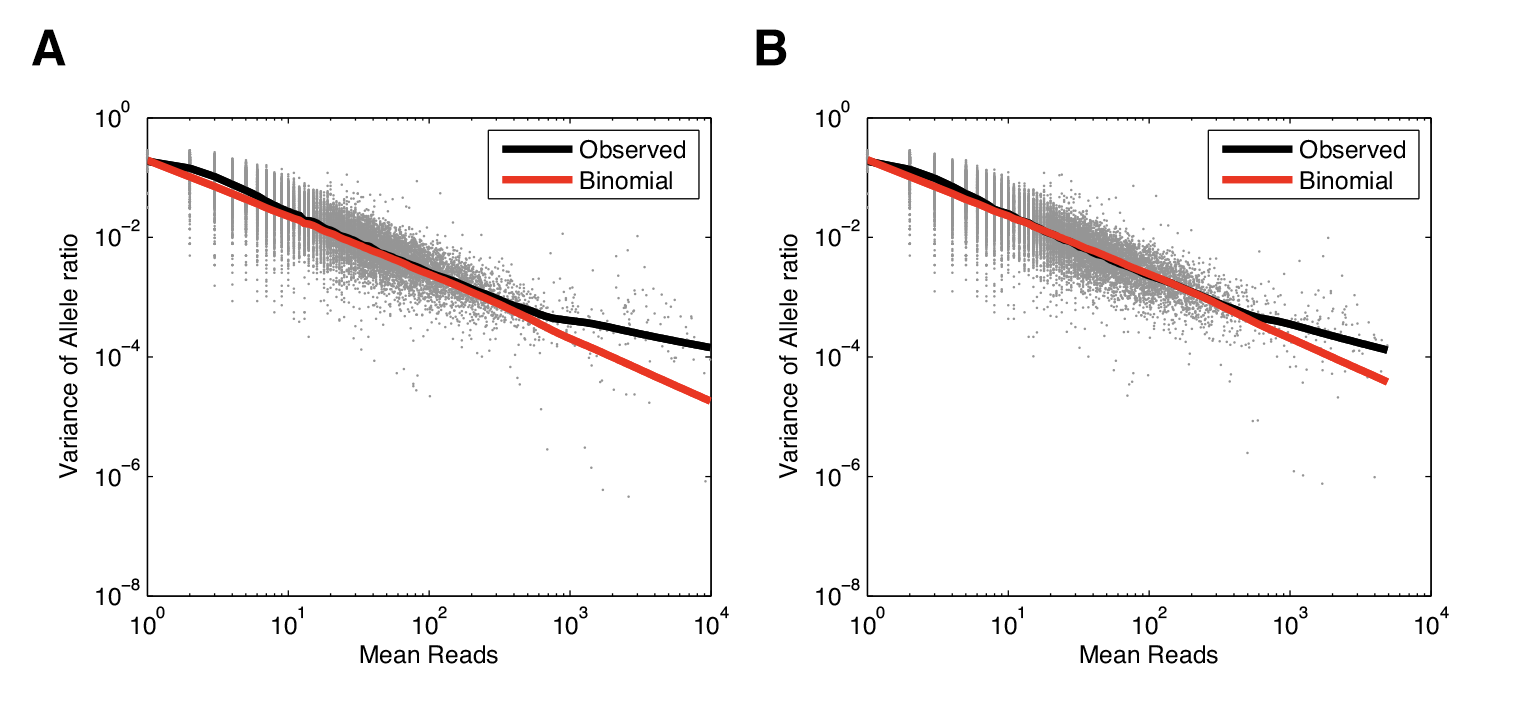

Supplement: Additional file 9: Figure S8. — QC measures reduce overdispersion in technical replicates when testing for allelic imbalance using a binomial test. Variance of allelic ratios as a function of total read counts, calculated as the mean at a given SNP from a Geuvadis individual with eight technical replicates (grey) with (b) or without (a) accounting for duplicate reads and overlapping read mates. The lines denote locally weighted smoothing of observed data (black) and theoretical variance for binomially distributed data (red). (TIFF 3209 kb) [file 13059_2015_762_MOESM9_ESM.tif]

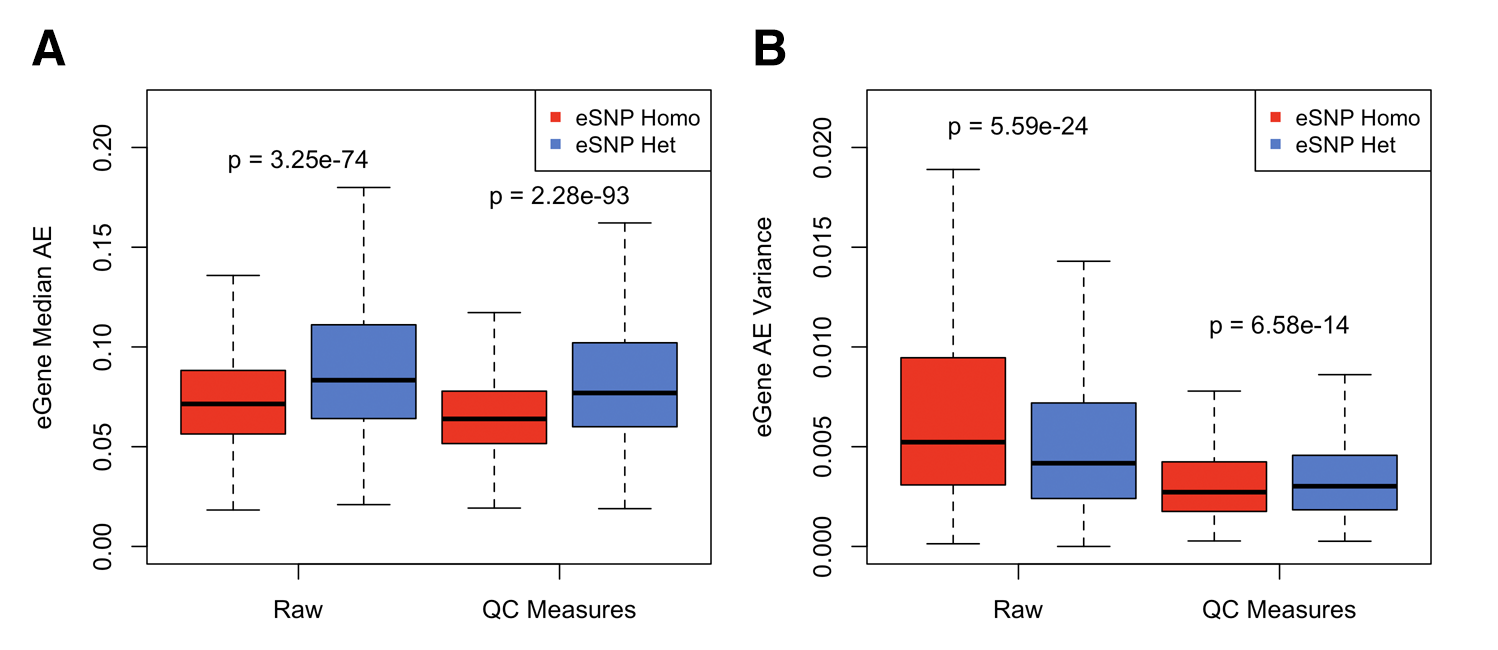

Supplement: Additional file 11: Figure S9. — QC measures improve the power to detect biologically relevant allelic expression at genes that have eQTLs (eGenes), where individuals that are heterozygous for the top eQTL SNP (eSNP) are expected to have more allelic expression than homozygous individuals (extended). a QC measures increase the significance of the difference between heterozygous and homozygous individuals within eGenes. b QC measures reduce the variance of allelic expression between individuals within eGenes. (TIFF 2856 kb) [file 13059_2015_762_MOESM11_ESM.tif]

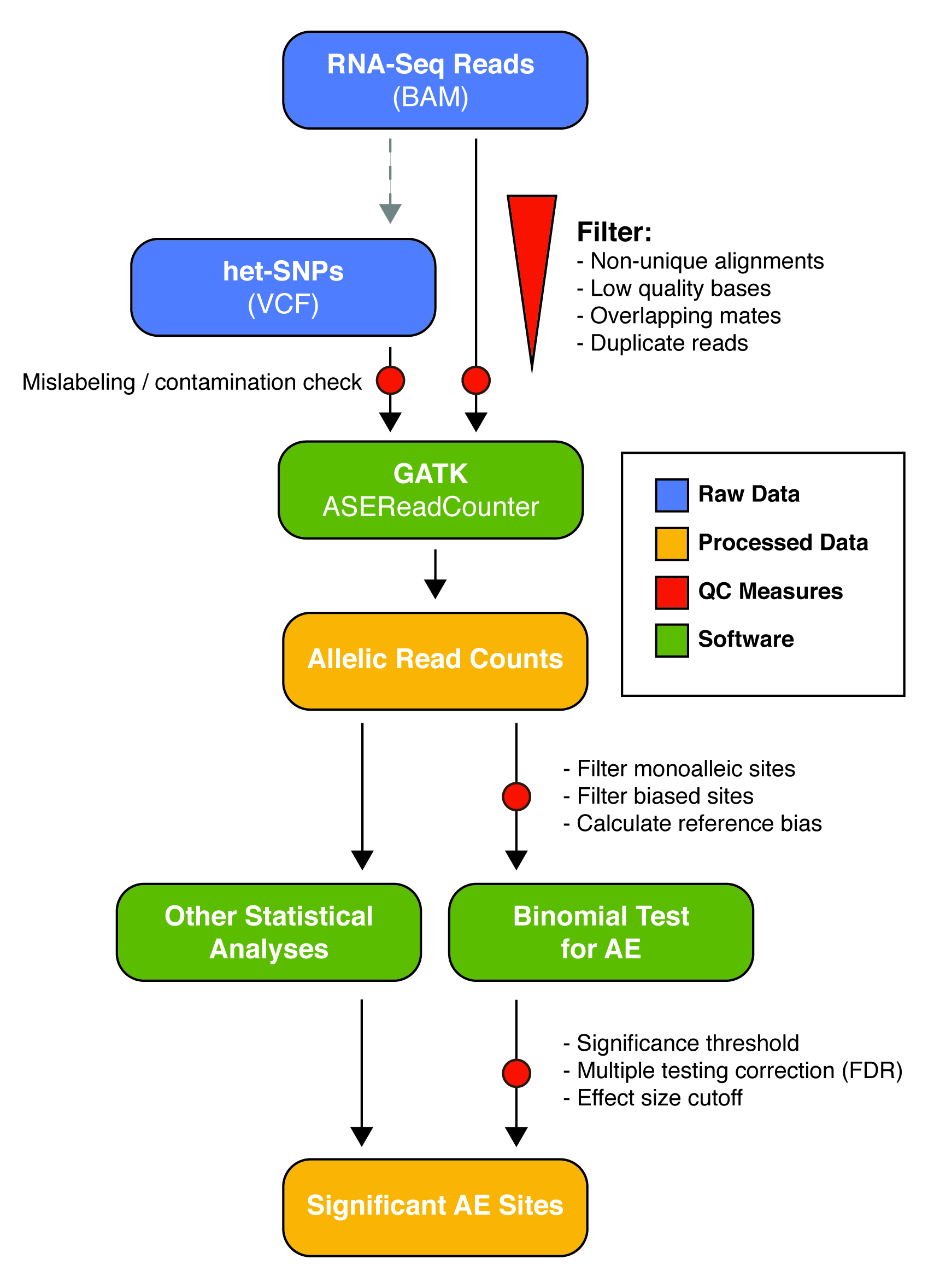

Supplement: Additional file 12: Figure S10. — Complete workflow for AE analysis illustrating appropriate quality control measures and filters. (TIFF 782 kb) [file 13059_2015_762_MOESM12_ESM.tif]
